# Pyridoxine for Prevention of Hand-Foot Syndrome Caused by Chemotherapy: A Systematic Review

**DOI:** 10.1371/journal.pone.0072245

**Published:** 2013-08-20

**Authors:** Min Chen, Lingli Zhang, Qian Wang, Jiantong Shen

**Affiliations:** 1 Department of Pharmacy, West China Second University Hospital, Sichuan University, Chengdu, China; 2 Key Laboratory of Birth Defects and Related Diseases of Women and Children (Sichuan University), Ministry of Education,Chengdu, China; 3 Evidence-Based Pharmacy Centre,West China Second University Hospital, Sichuan University, Chengdu, China; 4 West China School of Pharmacy, Sichuan University, Chengdu, China; 5 Department of Rehabilitation Medicine, West China Hospital, Sichuan University, Chengdu, China; 6 Chinese Cochrane Centre and Chinese Evidence-Based Medicine Centre, West China Hospital, Sichuan University, Chengdu, China; University of North Carolina School of Medicine, United States of America

## Abstract

**Background:**

Hand-foot syndrome (HFS) is a relatively frequent dermatologic toxic reaction to certain anti-cancer chemotherapies. The syndrome can evolve into a distressing condition that limits function and affects quality of life. Pyridoxine (vitamin B6) has been used empirically for the prevention of HFS caused by anti-cancer therapy. However, evidence of its efficacy remains controversial.

**Methodology//Principal Findings:**

Systematic literature searches were conducted on the Cochrane Library, PUBMED, EMBASE, LILACS, CBM, CNKI, VIP, WANFANG and the U.S. ClinicalTrials.gov website. We included all related randomized controlled trials (RCTs) irrespective of language. Reviewers from different professions independently assessed all potential studies and extracted data. Subgroup analysis was planned according to dose of pyridoxine. 5 RCTs involving 607 patients were contributed to the meta-analysis. No significant differences were found between patients receiving pyridoxine and placebo for prevention of incidence of HFS and grade 2 or worse HFS (relative risk (RR) 0.96, 95%confidence interval (CI) 0.86–1.06; RR0.95, 95%CI 0.73–1.24, respectively). Similarly, no significant improvement in quality of life was detected among patients. However, significant difference was found for prevention of grade 2 or worse HFS with pyridoxine 400 mg daily compared to 200 mg (RR0.55, 95%CI 0.33–0.92).

**Conclusions/Significance:**

There is inadequate evidence to make any recommendation about using pyridoxine for prevention of HFS caused by chemotherapy. However, pyridoxine 400 mg may have some efficacy. Further studies of large sample sizes are needed to evaluate the efficacy and safety of pyridoxine, especially at high dose, in comparison with placebo.

## Introduction

Hand-foot syndrome (HFS), also known as palmar-plantar erythrodysesthesia (PPE), palmar-plantar erythema, acral erythema and Burgdorf’s reaction, is a relatively frequent dermatologic toxic reaction to certain anti-cancer chemotherapies [1]. HFS has been reported in 6% to 42% of patients being treated with cancer chemotherapeutic agents, such as 5-fluorouracil (5-FU), doxorubicin, cytarabine, cyclophosphamide, vinorelbine, docetaxel or multikinase inhibitors sorafenib and sunitinib [2], [3].

The major clinical symptoms are typically described as a dermatologic reaction including erythema, swelling, twinge in the palms and soles [4]. The syndrome is usually mild, but can evolve into a distressing condition that limits function and affects quality of life (QoL). Although it is not a life threatening toxicity, HFS can be quite serious, resulting in dose reduction and shortened cancer treatment duration or intensity [5]. HFS toxicity is usually collected and scored according to the National Cancer Institute Common Toxicity Criteria for Adverse Events (NCI-CTCAE) by investigators and clinical research coordinators. In NCI-CTCAE version3.0, Grade 1 HFS is characterized by minimal skin changes or dermatitis without pain; grade 2 is characterized by skin changes or pain, but not interfering with function and grade 3 is characterized by ulcerative dermatitis or skin changes with pain interfering with function [6].

The pathogenesis of HFS is unclear to date [4]. Numerous approaches have been employed in attempts to prevent and/or reduce the incidence of HFS. Drug-related therapies include topical emollients and creams, systemic and topical corticosteroids, pyridoxine (vitamin B6), nicotine patch, vitamin E and COX-2 inhibitors [7]. As a relatively nontoxic and inexpensive treatment, pyridoxine has been used empirically for the prevention of HFS caused by antineoplastic chemotherapies [8], [9].

In recent years, many institutions such as the UK Addenbrooke's Hospital, the USA Thomas Hospital, the Korea University of Ulsan College of Medicine, the Thailand Chulalongkorn University, the China Luoyang Dongfang Hospital and the Singapore National Cancer Centre are ongoing or have completed randomized controlled trials to detect how well pyridoxine works in patients with chemotherapy [10], [11], [12], [13], [14], [15]. However, evidence of its efficacy remains controversial. Therefore, we performed a systematic review to evaluate the efficacy of pyridoxine which was administered for prevention or treatment of HFS in anti-cancer therapy.

## Methods

### Ethics Statement

The study was approved by the medical ethics committee of west china second university hospital.

### Searching

We searched Cochrane Central Register of Controlled Trials (CENTRAL) published in Cochrane Library (2013, Issue 1), PUBMED, EMBASE using the search strategy detailed in table S1; we searched Chinese Biomedical Literature Database (CBM) and Chinese National Knowledge Infrastructure (CNKI); VIP Database for Chinese Technical Periodicals (VIP) and WANFANG for literatures published in Chinese. We also searched LILACS and the U.S. ClinicalTrials.gov website with the search term pyridoxin*, vitamin B6, hand foot syndrom* and palmar-plantar erythrodysesthesia. The references of all retrieved articles were scanned for additional relevant citations. We searched all databases from their earliest records to February 2013.

### Eligibility Criteria

Randomized controlled trials published in full text or abstract only were both included, and there was no restriction on publication language. Eligibility criteria included adult patients older than 18 years receiving anti-cancer chemotherapies; Eastern Cooperative Oncology Group performance status 0–2; life expectancy more than 12 weeks and no contraindication to chemotherapy (i.e., adequate bone marrow function and normal renal and liver function). Exclusion criteria included previous treatment for HFS; hypersensitivity to pyridoxine; pregnancy or lactation. The intervention was pyridoxine (vitamin B6) regardless of the dose and duration, compared with placebo or no treatment. Studies that enrolled combination of drug use for HFS were also excluded.

### Study Selection and Management

Reviewers from different professions independently assessed all potential studies and extracted data. Two authors (MC and QW) independently screened the title, abstract and key words of all studies identified by the search strategy and obtained the full articles for all potential relevant trials. Three authors(MC, QW and JTS)independently assessed the full text and extracted data using a data extraction form. Disagreement was resolved by discussion or consulting with the corresponding author (LLZ).

For dichotomous outcomes, we extracted the number of participants experiencing the event and the total number of participants evaluated for that outcome. For continuous outcomes, we extracted the mean, standard deviation (SD) or any data which could be used to derive the SD, and the total number of participants evaluated for that outcome [16]. Subgroup analysis was planned according to dose of pyridoxine.

### Risk of Bias Assessment

Three authors(MC, QW and JTS) independently assessed the risk of bias using a standard form. We contacted the authors by phone or email if important information was unclear. We used the domain-based evaluation recommended by the Cochrane Handbook (Higgins 2011) to address six specific domains: sequence generation, allocation sequence concealment, blinding, incomplete outcome data, selective outcome reporting and other potential sources of bias [16].

### Statistical Synthesis

As a measure of effectiveness, risk ratio (RR) with 95% confidence interval (CI) was calculated for the meta-analysis. We determined the presence of heterogeneity using I^2^ statistic and used the fixed-effect model to combine trials in the absence of substantial heterogeneity (I^2^<50%). By contrast, random-effect model was used when heterogeneity was significant (I^2^≥50%) and could not be explained by subgroup analysis, clinical or methodological features of the trials. Meta-analysis was performed using Review Manager Version5.1.0 software [17].

## Results

### Study Selection

**Figure 1:**
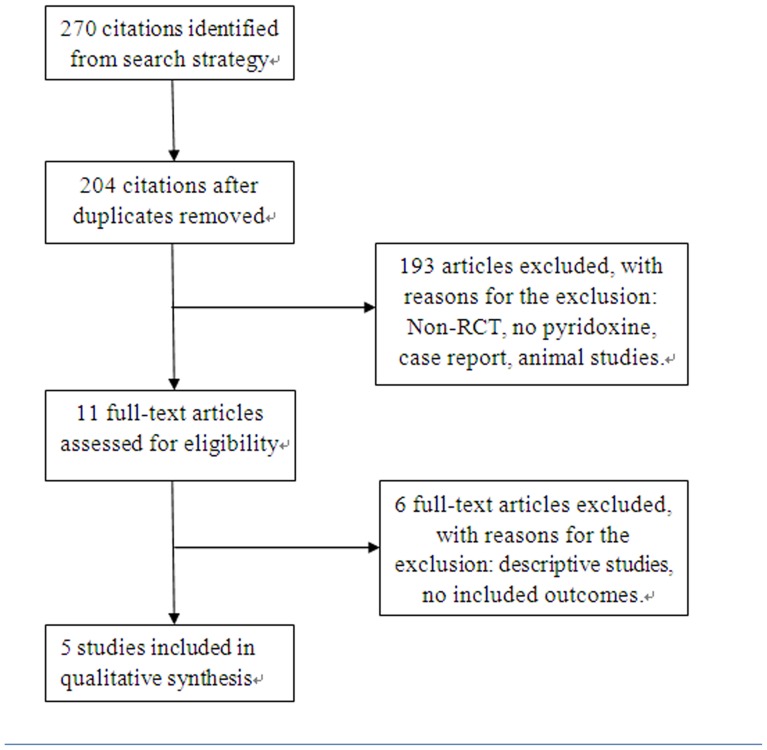
Flow diagram of study selection process. This PRISMA 2009 flow diagram illustrates the results of search and the process of screening and selecting studies for inclusion, and the reasons for exclusions in this review.

Figure 1 showed the literature selection process. Based on the search strategy we identified 270 articles, of which 259 were excluded by the reviewers after reading the titles and abstracts. 11 relevant full articles were read and only 5 studies were included eventually [10], [11], [12], [13], [14].

### Characteristics of Included Studies

Table 1 provided details for each trial. All studies described their hypothesis/objective and main findings clearly. 612 patients were included and the mean age was 62.1 years (range from 20–87). A wide range of cancer types including colorectal, breast, ovarian, stomach, biliary tract, endometrial, duodenum was represented, with colorectal the most common (350 patients). The capecitabine dose was initiated at 2000–2500 mg/m^2^ orally per day for alone or combined treatment, for 2 weeks followed by 7 days rest. The Pegylated Liposomal Doxorubicin dose was 40 mg/m^2^ intravenously every 4 weeks for single-agent therapy. The pyridoxine was prescribed orally commencing the same day that chemotherapy was initiated. Treatment continued until disease progression, toxicity or patient preference. 5 patients were excluded because of reactions to chemotherapy during the first course. The remaining 607 patients were contributed to the meta-analysis.

**Table 1:** Characteristics of the included studies.

study	country	patients					Interventions		outcomes
		number	age average	gender	cancer type	chemotherapy drug	T	C	
Chalermchai 2010[^13^]	Thailand	56	56.7(range,26–82)	female(57%) male(43%)	breast cancer:17 colorectal cancer:39	capecitabine	400 mg/d po	200 mg/d po	(2),(3),(4),(5)
Corrie 2012[^10^]	UK	106	73(range,42–87)	female(64%) male(36%)	colorectal cancer:68 breast cancer:38	capecitabine	150 mg/d po	placebo	(1),(2),(5),(6),(7),(8)
Fang 2010[^14^]	China	56	61(range,38–78)	female(48%) male(52%)	breast cancer:18 colorectal cancer:28 stomach cancer:10	capecitabine	300 mg/d po	-	(1),(2)
Gruenigen 2010[^12^]	USA	34	64(range,45–81)	female(100%)	ovarian/peritoneal cancer:25 endometrial cancer:8 breast cancer:1	pegylated liposomal doxorubicin	200 mg/d po	placebo	(1),(2), (6), (9)
Kang 2010[^11^]	Korea	360	56(range,20–75)	female(37.5%) male(62.5%)	stomach cancer:132 colon cancer:215 biliary tract cancer:12 duodenum cancer:1	capecitabine	200 mg/d po	placebo	(1),(2),(4)
Total		612	62.1(range,20–87)						

Abbreviation: T, treatment group; C, control group; –, no treatment; po. per os.

Outcomes reported:(1)Incidence of all grades HFS; (2)Incidence of grade 2 or worse HFS; (3)Time to development of grade 2 or worse HFS; (4)Factors affecting development of HFS;(5)Tumor response;(6)Quality of life;(7)Chemotherapy drug dose modification;(8)Progression-free survival;(9)Incidence of Adverse Events excluding HFS.

### Risk of Bias Assessment in Included Studies

**Figure 2:**
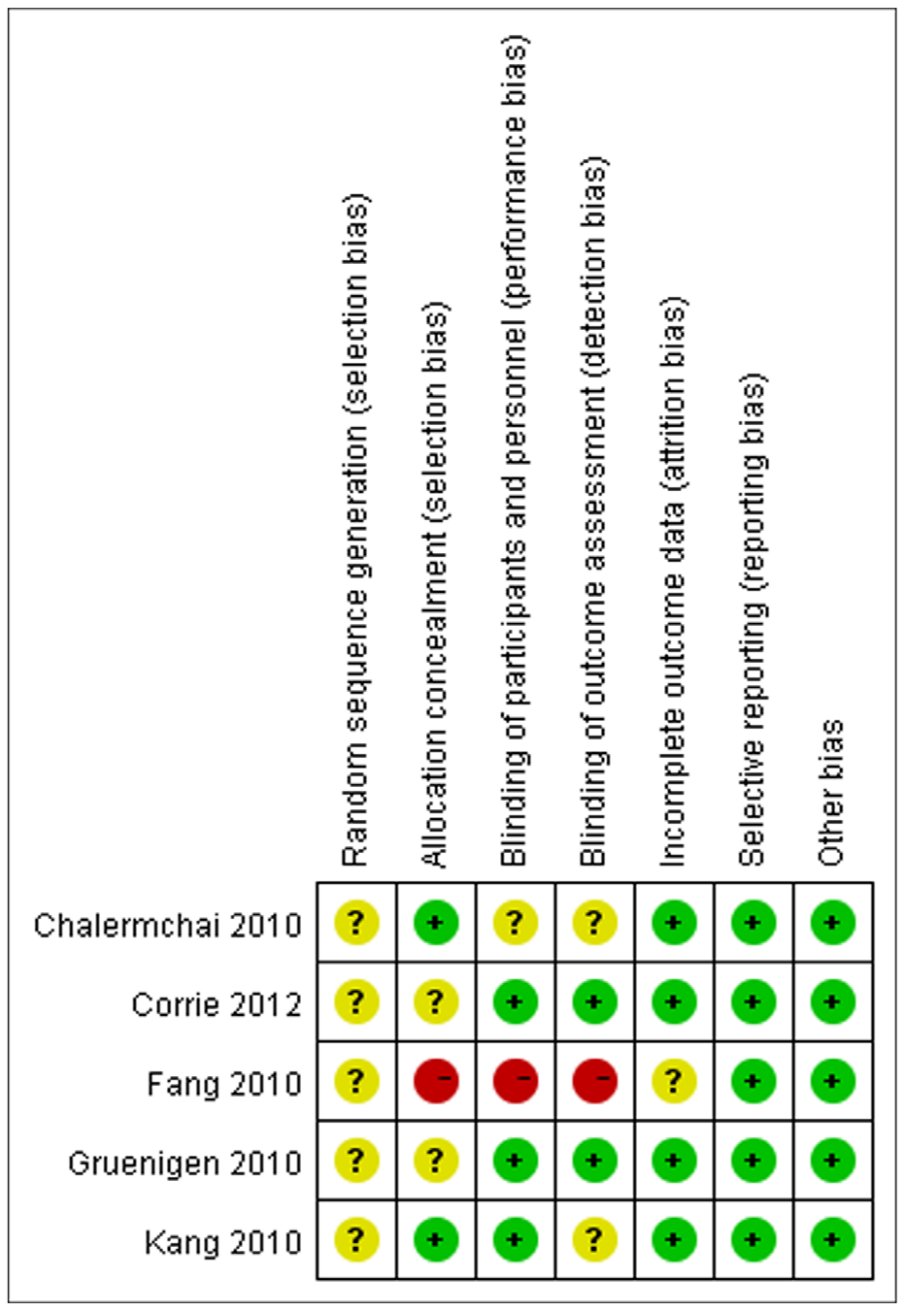
Quality assessment in included studies. This plot is created by the software of RevMan 5.1.0. It illustrates the quality of included studies with each of the judgement (‘low risk’, ‘high risk’ or ‘unclear risk’ of bias). All studies had low risk bias in selective reporting and other issues, and unclear risk in random sequence generation. One study (Fang 2010) had high risk bias in allocation concealment and blinding.

Figure 2 provided methodological details for each trial. None of the studies provided specific random sequence generation although they described randomized design. All studies had low risk bias in selective outcome reporting and other potential issues. However, one trial [14] had high risk bias in allocation concealment and blinding which was implemented by sensitivity analysis.

### Incidence of HFS

**Figure 3:**
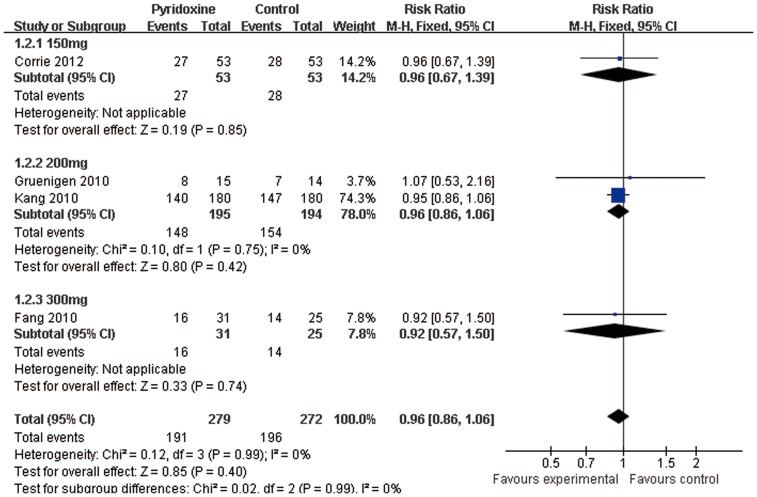
Forest plot showing the meta-analysis of pyridoxine versus placebo in the incidence of Hand-foot syndrome. This forest plot is created by the software of RevMan 5.1.0. Horizontal lines indicate 95% CIs. Solid boxes indicate the response rate in each study. Test of heterogeneity (I^2^ = 0%) indicates the absence of substantial heterogeneity. The bottom of diamond indicates the pooled response rate (RR0.96, *P* = 0.99).

Four trials [10], [11], [12], [14] reported oral pyridoxine versus placebo in the incidence of HFS. We did not find any statistically significant difference in the incidence of HFS among patients receiving placebo compared to oral 150 mg daily of pyridoxine (relative risk (RR) 0.96; 95%confidence interval (CI) 0.67–1.39; n = 106) and oral pyridoxine 200 mg (RR0.96; 95%CI 0.86–1.06; n = 389) and oral pyridoxine 300 mg (RR0.92; 95%CI 0.57–1.50; n = 56). Totally, there were no statistically significant differences in the risk of HFS among patients receiving placebo compared to pyridoxine (RR0.96; 95%CI 0.86–1.06; n = 551) (Fig.3).

### Incidence of Grade 2 or Worse HFS

We planned subgroup analysis according to dose of pyridoxine.

#### Pyridoxine versus Placebo

**Figure 4:**
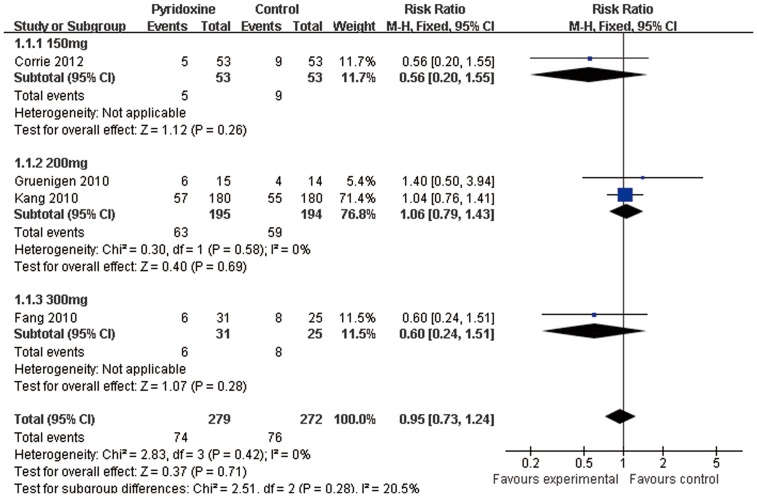
Forest plot showing the meta-analysis of pyridoxine versus placebo in the incidence of grade 2 or worse Hand-foot syndrome. This forest plot is created by the software of RevMan 5.1.0. Test of heterogeneity (I^2^ = 0%) indicates the absence of substantial heterogeneity. The bottom of diamond indicates the pooled response rate (RR0.95, *P* = 0.28).

Four trials [10], [11], [12], [14] reported oral pyridoxine versus placebo in the incidence of grade 2 or worse HFS. We did not find any statistically significant difference among patients receiving placebo compared to oral 150 mg daily of pyridoxine(RR0.56; 95%CI 0.20–1.55; n = 106) and oral pyridoxine 200 mg (RR1.06; 95%CI 0.79–1.43; n = 389) and oral pyridoxine 300 mg (RR0.60; 95%CI 0.24–1.51; n = 56). Totally, there were no statistically significant differences in the risk of grade 2 or worse HFS among patients receiving placebo compared to pyridoxine(RR0.95; 95%CI 0.73–1.24; n = 551)(Fig.4).

#### Different Doses of Pyridoxine

**Figure 5:**
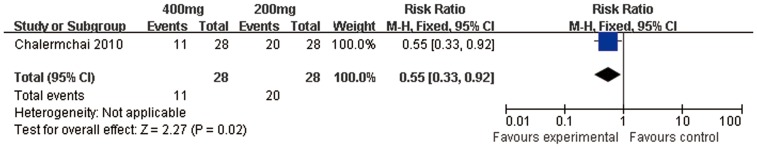
Forest plot showing the meta-analysis of pyridoxine 400 mg versus 200 mg in the incidence of grade 2 or worse Hand-foot syndrome. This forest plot is created by the software of RevMan 5.1.0. The diamond indicates the response rate (RR0.55, *P* = 0.02).

One trial [13] reported oral pyridoxine 400 mg versus 200 mg in the incidence of grade 2 or worse HFS. Pyridoxine 400 mg was more effective in the prevention of grade 2 and grade 3 HFS than pyridoxine 200 mg (RR 0.55; 95% CI 0.33–0.92; n = 56) (Fig.5).

### Time to the Development of Grade 2 or Worse HFS

One trial [13] reported the median time to the development of grade 2 or worse HFS in pyridoxine 400 mg group (87 days) was slightly longer than 200 mg group (61 days). However, we did not find any statistically significant difference (*P* = 0.44).

### Quality of Life

Two trials [10], [12] evaluated the QoL between the pyridoxine and placebo groups. There were no significant differences between the two groups in the QoL no matter in 106 patients treated with capecitabine using the European Organization for Research and Treatment of Cancer QLQ-C30 instrument or 34 patients treated with pegylated liposomal doxorubicin using FACT-General instrument. Both of the instruments were used to assess the impact of HFS on QoL.

### Sensitivity Analysis

We performed sensitivity analysis after exclusion of one RCT with low quality. After exclusion of the trial evaluating the use of pyridoxine in the prevention of HFS and grade 2 or worse HFS, Meta-analysis showed that the differences were not significant (RR0.96, 95% CI0.86–1.07; RR0.99, 95% CI0.75–1.32; respectively) (Table 2).

**Table 2:** Results of Sensitivity Analyses(fixed-effect model).

Strata of Sensitivity Analysis Results for Each End Point	RCTs	RR(95%CI)	*P*	Heterogeneity(*P*)
Incidence of HFS	[10], [11], [12]	0.96(0.86–1.07)	0.43	NS
Incidence of grade 2 or worse HFS	[10], [11], [12]	0.99(0.75–1.32)	0.97	NS

Abbreviation: NS, not significant.

## Discussion

Pyridoxine has been used frequently for HFS associated with chemotherapy. However, the mechanism by which pyridoxine protects against HFS is still not fully understood. In the patient’s hand, punch biopsy results showed vacuolar degeneration of the basal layer of the epidermis with cellular enlargement, spongiosis, mild exocytosis of small lymphocytes, and marked hyperkeratosis [1]. According to the metabolism of pyridoxine, it could be converted into pyridoxal phosphate in red blood cells. And pyridoxal has been discovered as a potent antagonist of P2X purinergic receptor, which accelerates repair of the skin barrier and prevents epithelial hyperplasia [18].

Pyridoxine has been used successfully at dose of 50 to 800 mg/day for treating and preventing fluorouracil-, docetaxel-, etoposide-, doxorubicin- and sorafenib-related PPE [19], [20], [21], [22], [23], [24], [25], [26]. In a case report, pyridoxine 100 mg three times daily was used successfully to treat PLD-related HFS [27]. A small study in 25 metastatic colorectal cancer patients showed that prophylactic pyridoxine at 50 or 150 mg daily might be useful in delaying the onset of severe PPE from fluorouracil [20]. And a double-blind clinical trial using a canine model proved the efficacy of pyridoxine in delaying the onset and severity of PPE during doxorubicin containing pegylated liposome chemotherapy [28]. Although pyridoxine has been shown to be effective, a negative effect on response duration was reported when pyridoxine was given at 300 mg/m^2^ to prevent hexamethylmelamine-related neurotoxicity [29].

Based on the current evidence, this is the first systematic review of five randomized controlled studies that estimates the efficacy of pyridoxine for the prevention or treatment of HFS. Results revealed that there was inadequate evidence to support the use of pyridoxine in the prevention of HFS caused by chemotherapy. No statistically significant differences were found among patients receiving pyridoxine (150 mg, 200 mg, 300 mg) compared with placebo. Moreover, the two sensitivity analyses showed similar results. Although pyridoxine was not preventative at 150–300 mg daily, it might be beneficial at high dose. Chalermchai et al (2010) suggested that high dose of pyridoxine 400 mg was more effective than 200 mg in the protection for incidence of grade 2 or worse HFS. Nevertheless, it was noteworthy that when the endpoint was incidence of grade 3 HFS or the time to onset of grade 2 or worse HFS in this RCT, no statistically significant differences were found between 400 mg and 200 mg groups. Therefore, the study results might be limited by small sample size.

In this review, another observation showed that pyridoxine had no meaningful impact on the quality of life in patients with anti-cancer therapy. In addition, all the studies eligible failed to compare effectiveness in the time to development of HFS, chemotherapy drug dose modification, progression-free survival, incidence of adverse events excluding HFS among pyridoxine and control groups.

Our study has several limitations. First, information from primary studies was not sufficient to perform subgroup analysis by types of chemotherapy regimen. To our knowledge, the frequency of HFS in patients differs between various medical tumor therapies. It has been reported in 50–60% of patients being treated with capecitabine and 22–26% with doxorubicin [5]. Besides, this review only included randomized controlled trials in which adverse effects of pyridoxine were not assessed absolutely. All of these need improvement in future studies.

## Conclusions

Based on the data from five randomized controlled trials, there is inadequate evidence to make any recommendation about using pyridoxine for prevention of HFS caused by chemotherapy. However, pyridoxine 400 mg may have some efficacy. Further studies of large sample sizes are needed to evaluate the efficacy and safety of pyridoxine, especially at high dose, in comparison with placebo.

HFS is a common health problem among patients with chemotherapy. And any treatment which might prove to be effective is worth investigation.

## Supporting Information

Table S1 **Search strategy for PUBMED, CENTRAL and EMBASE.**
(DOC)

Table S2 **PRISMA checklist applied to this review.**
(DOC)
