# Supplementary material for: Pyridoxine for Prevention of Hand-Foot Syndrome Caused by Chemotherapy: A Systematic Review
Source: PLoS One. 2013 Aug 20;8(8):e72245. doi: 10.1371/journal.pone.0072245 (PMC3748123; doi:10.1371/journal.pone.0072245)
Supplement: Table S1 — Search strategy for PUBMED, CENTRAL and EMBASE. (DOC) [file pone.0072245.s001.doc]

**Table S1: Search strategy of databases**

| Databases | Search Strategy |
| --- | --- |
| PUBMED | #1 vitamin B6[mesh] OR pyridoxin*[tiab] OR pyridoxol [tiab] OR vitamin B6[tiab] |
|  | #2 Hand-Foot Syndrome[mesh] OR hand foot syndrom*[tiab] OR palmoplantar erythrodysesthesia*[tiab] OR palmoplantar erythema* [tiab] OR acral erythema*[tiab] OR Burgdorf’s reaction[tiab] OR hand–foot skin reaction[tiab] |
|  | #3 #1 and #2 |
| CENTRAL | #1 vitamin B6[MeSH descriptor: explode all trees] OR pyridoxin*[tiabkw] OR pyridoxol [tiabkw] OR vitamin B6[tiabkw] |
|  | #2 Hand-Foot Syndrome[MeSH descriptor: explode all trees] OR hand foot syndrom*[tiabkw] OR palmoplantar erythrodysesthesia*[tiabkw] OR palmoplantar erythema*[tiabkw]OR acral erythema*[tiabkw]OR Burgdorf* reaction[tiabkw] OR hand–foot skin reaction[tiabkw] |
|  | #3 #1 and #2 |
| EMBASE | #1 pyridoxine[emtree] OR pyridoxin*[tiab] OR pyridoxol [tiab] OR vitamin B6[tiab] |
|  | #2 Hand Foot Syndrome[emtree] OR hand foot syndrom*[tiab] OR palmoplantar erythrodysesthesia*[tiab] OR palmoplantar erythema*[tiab] OR acral erythema*[tiab] OR Burgdorf* reaction[tiab] OR hand–foot skin reaction[tiab] |
|  | #3 #1 and #2 |

Abbreviation: ti,title; ab,abstract; kw,keyword
